# Groundnut spread likability, sensory properties, and intent to pay for quality certification

**DOI:** 10.29219/fnr.v64.3600

**Published:** 2020-01-09

**Authors:** Ozgur Kaya, Wojciech J. Florkowski, Daniel Sarpong, Manjeet S. Chinnan, Anna V. A. Ressurrecion

**Affiliations:** 1 Department of Economics, School of Business Administration, American University of Sharjah, Sharjah, United Arab Emirates; 2 Department of Agricultural and Applied Economics, University of Georgia, Griffin Campus, Griffin, GA, USA; 3 Department of Agribusiness Management, University of Ghana-Legon, Legon, Ghana; 4 Department of Food Science and Technology, University of Georgia, Griffin, GA, USA

**Keywords:** Groundnut, quality certification, untrained panel, likability, aroma, aflatoxin awareness

## Abstract

Quality-certified, nutritious novel groundnut spread has great commercialization possibilities due to evolving urban lifestyles in Africa, but lack of information about likability, sensory attributes, and consumer safety awareness is a severe barrier for small enterprises.

This paper examines a novel groundnut spread, made of sorted kernels deemed free of aflatoxin, intended for use on bread in a fashion similar to groundnut paste or groundnut butter, but with modified sensory characteristics. In particular, it seeks to measure the effects of sensory attributes of the novel spread on the intent to pay for safety certification and the role of consumer awareness of aflatoxin.

A novel spread was prepared with groundnut paste from sorted kernels (to eliminate the risk of aflatoxin contamination) and cocoa. Adults intercepted at Ghana’s International Fair in 2012 volunteered to sample the spread and complete a questionnaire. Results from a tasting panel of untrained participants established that sensory attributes and panellist characteristics are relevant to the intent to pay for quality certification. Spread likability, aroma, education, knowledge about aflatoxin, packaging and being married were identified as major factors increasing the probability of intent to pay for quality certification whereas young age and the presence of children in a household lowered the probability. Results also identified income, education level, and having young children at home as increasing the chances of knowing about aflatoxin.

Groundnut paste available in Ghana is often contaminated by aflatoxin as it is in other countries in the region and consumers cannot visually assess paste quality. Under the circumstances, quality certification is necessary.

## Popular scientific summary

- Urban lifestyles, rising incomes, nutrition, and quality awareness drive development of novel spread.
- Domestic food manufacturers can offer a quality-certified groundnut spread that promises success in marketplace by using local ingredients.
- Spread likability and aroma are major factors behind intent to pay for quality certification.
- Quality certification payment intent is higher among educated and married people, while it is lower among young and panelists with children.

Groundnuts are a relatively inexpensive protein source produced by African farmers and well-liked by consumers. Traditional ways of eating groundnuts involve the use of groundnut paste as an ingredient to various dishes, including soups stews, and sauces (1, 2), but nowadays groundnut paste is also consumed as a spread on bread and other baked foods. The use of groundnut paste as a spread is relatively new and practiced more often by Ghanaian urban consumers, who, similar to many Africans, have gradually departed from the traditional diet (3) as this form of consumption fits the evolving urban lifestyle (4). The combination of groundnut protein and cereal has been suggested as the way to improve the protein and nutrient density in African diets (5) and to combat malnutrition in societies (4).

With consumer incomes increasing in Ghana, the opportunity arises to develop and promote novel foods based on domestically available ingredients. Consumers are interested in functional food products, and groundnuts are a functional food (6–8). However, food processors have little knowledge of consumer preferences, including product taste and preferences (9, 10). The novelty associated with the modification of an existing food product enhances its public understanding and its ingredients and assures acceptability. A groundnut paste with the addition of domestically produced cocoa powder has been used to develop a novelty: a chocolate spread intended for use on bread or bakery products in Ghana. The spread has two ingredients (cocoa and groundnut) with bioactive components creating functional food product, a good source of protein, fatty acids, and important micronutrients.

The disadvantage of groundnuts as a protein source is their susceptibility to contamination by aflatoxin, a metabolite of *Aspergillus* spp. Ingestion of aflatoxin leads to severe health problems, and various procedures have been recommended to reduce aflatoxin presence (11). The emerging food manufacturers in Ghana could refer to sorting as a feasible method of assuring consumers that the groundnut product is aflatoxin-safe and establish a claim to label a product as quality certified. Novelty-seeking consumers who pay attention to food safety will purchase the quality-certified product, especially if the product is well-liked. Choosing the novel spread shows preferences for quality, but the cost of certification has been viewed as a limiting factor (9), while sorting kernels implies that a portion of kernels is removed and represents a loss to a processor. The loss of potential raw material has to be recouped by processor to assure economic viability of the firm.

This article examines novel groundnut spread, made from sorted kernels deemed free of aflatoxin, intended for use on bread in a manner similar to groundnut paste or groundnut butter, but with modified sensory characteristics. The addition of cocoa and the safety certification will differentiate the product from a plain groundnut paste commonly found in Ghana. In particular, the study seeks to measure the effects of sensory attributes of the novel spread on the intent to pay for safety certification and the role of consumer awareness about aflatoxin. There is no scientific published study on the role of attributes of that type of spread that help predict the consumer decision in countries located in sub-Saharan Africa. Since the majority of food processing companies are small, owned by female entrepreneurs in Ghana (12–14), knowledge generated by the study offsets the gap in understanding the link between buyer preferences for sensory attributes and product safety assurances, typically limiting their market success (9, 10).

## Materials and methods

### Samples

Shelled groundnuts used to manufacture the spread were purchased from an open-air market in Accra agglomeration. Kernels were sorted on the processor premises using the recommended manual sorting method (15). In contrast to developed economies, groundnuts traded in Ghana are not identified by variety but rather by their appearance, primarily the color of testa and kernel size. A type of groundnuts commonly used for making groundnut paste are small-kernel groundnuts, called ‘Chinese’ by traders, resembling the ‘Florunner’ variety. This type differs from the large kernel ‘Bugla’ used mostly to make roasted groundnuts or another type (‘manipintar’) characterized by high oil content and initially introduced by the government to produce groundnut oil. None of the traded types is genetically uniform because they are grown from seeds saved by farmers and traded at regional markets, while certified variety-specific seeds were not available.

The process of manual sorting involves the removal of testa (blanching), and this step differs from traditional processing of groundnuts into paste in Ghana during which testae are left in the batch. Blanching allows the visual inspection of kernels and the removal of visibly damaged kernels or splits. Once sorted, kernels are roasted to develop the flavor. The type of groundnuts used is important because groundnut germplasm and variety effect the taste of roasted groundnut (4), and also because the uniformity of kernel size assures even roasting. Roasted kernels were ground in a plate mill, a commonly available equipment in mills providing grinding services in Ghana. Later, cocoa and other ingredients were added to obtain the final product, the groundnut–choco spread.

### Sensory testing and panelist information

To test how well a product is liked, this study applies the hedonic or affective type of test from among the available sensory evaluation methods (16). This method implies the use of untrained panel screened for product use. In the case of groundnut spread, the data collection consisted of two parts. The untrained panel (naïve panel) was chosen based on the availability and interest from among the visitors at Ghana’s International Fair in Accra in July and August 2012. The panelists were likely representatives of the consumers who are familiar with groundnut paste and capable to indicate the level of acceptability of its sensory attributes, while their personal and household features are consistent with the ability to purchase a novel spread. The testing took place throughout the duration of the fair at the booth operated by the small food processing company. Organization of product testing in a well-controlled environment remains prohibitively expensive for small food processors even if it is available in the country.

Each visitor who volunteered to participate in the test was offered a teaspoon of the spread followed by an opportunity to complete a questionnaire. None of the individuals who tested the spread was obligated to participate in the second part of the data collection. The questionnaire was self-administered. Previous studies have indicated that naïve panelists may have difficulty in describing their sensory experience (17), and hence a list of the sensory attributes included in the posed questions facilitated that task. Since the study focused on likability of the spread, the question probed for that specific attribute using a nine-point balanced scale with the centered neutral category (16). The equal distance between the points is amenable to regression analysis applied later in the current study.

Liking is the interaction of the consumer and the product (18). Lawless and Heymann note that consumers, such as those in Ghana who are familiar with groundnut paste commonly used in cooking, render opinions based on the integrated pattern of perceptions (16). Since the purpose of the study includes learning about the intention to pay for quality certification, knowing about the potential relevance of specific attributes expressed through nonanalytical spontaneous opinions is valuable. Additionally, a separate question focused on spread attributes and included a request to rate the importance of the spread taste, color, thickness and crunchiness using a five-point balanced scale (where 1 = not important at all to 5 = very important). These attributes play an important role in consumer appeal and in nut-spread buying decision (18), and the untrained panelists easily understood the applied terms describing attributes. Taste, experienced only during the sampling of the spread, is an essential attribute for sustained marketing and repeated sales, which are the ultimate goal of the food processor. Its role in the case of groundnut–choco spread is particularly important because the cost of safety certification increases the total cost and is later reflected in the price of marketed product. Color of the spread could matter because of the cocoa addition to the groundnut paste and the absence of a comparable product on the market at the time of the test. Color of groundnut paste can vary across varieties (4), but the novelty of adding cocoa restricted the ability of comparisons made by the untrained panelists, although negative perception of the color could create an obstacle to marketing. Color was found relevant in previous sensory studies (19), but its importance needs to be verified because it is specific for each spread. Thickness of the spread is relevant because the panelists, familiar with groundnut paste, likely compare that attribute to the novel product. Crunchiness is not typically expected in spreads, but the type of equipment used by small food processors (a plate mill) in Ghana yields groundnut paste that is commonly gritty and could be crunchy. Particle size in groundnut butter has been researched (20), but panelists are accustomed to locally accessible products, which are made using available equipment, and the perceptions are inherently subjective. A question of practical implications probed panelists about their preferred jar size for the novel spread offering several size choices. A common size of a jar visible in food stores in Ghana was the 450-g jar and reflected a common preference for a relatively large size container among price-sensitive consumers. That size plastic jars with twisted lid were commonly available for food processors and could be easily purchased from local suppliers.

Likability and other attributes of the spread do not exclusively determine the purchase of a novel spread. The apprehension about health effects leads to loss of trust and weakens marketing of nut spreads (18). Subsequently, other questions in the survey instrument probed the panelists for their views about intent to pay for quality certification of groundnut spread and awareness of aflatoxin. The questions required a straightforward choice of ‘yes’ or ‘no’, leading to creation of a binary variable used in statistical analysis.The panelists also shared information about personal and household characteristics pertinent to the objective of the study and used in regression analysis to generate their measurable effects on the probability of choosing to pay for quality certification in the context of knowing about aflatoxin, a major, internationally recognized food safety issue (21).

### Statistical method

Responses to questions about the intent to pay for quality certification of a groundnut spread and awareness of aflatoxin in foods, converted to numerical measures, have been coded as one of two possible outcomes reflecting the choice of ‘yes’ (1) or ‘no’ (0). When the dependent variable Y is binary, the population regression function is the probability that Y = 1, conditional on the regressors. The logit and probit regressions are nonlinear and specifically designed for estimation of equations with binary-dependent variables of the following nature:

Let y^*^ be an unobserved, or *latent*, variable, determined by

y*= *x β* + ε,

*y* = 1 if y* > 0,

*y* = 0 if y*1≤ 0,

where *x* is the explanatory variable vector, *β* is the coefficient vector, and is the random error term. Also, it is assumed that ε is independent of *x* and that e has either the standard logistic distribution (logit model) or the standard normal distribution (probit model). There may be practical reasons for favoring logit or probit in some cases, but it is difficult to justify the choice on theoretical grounds (22). Therefore, the choice is based on a comparison of test results. Accordingly, based on the Akaike’s Information Criterion (AIC) and the Bayesian Information Criterion (BIC), the logistic equations produced smaller values of both tests than the alternative probit equations and were applied in the current study.

The evaluation of goodness-of-fit of logistic equations may involve several formulas of a pseudo *R*^2^. The current study reports McFadden’s *R*^2^, a measure most commonly used in empirical studies. When comparing two equations applying the same data, the McFadden’s *R*^2^ would be higher for the equation with the greater likelihood (23). Additionally, this study reports Tjur’s coefficient of discrimination, that is, Tjur’s *R*^2^ (24).

## Results

### Panel summary results

Product features are essential, especially in a novel product in spite of containing the well-known and accepted groundnut paste and cocoa. A question listing five attributes of groundnut paste aimed at collecting information about the relative importance of each attribute was included in the survey. More than four out of five (83%) panelists liked the groundnut–choco spread, and the mode value was 9 on the nine-point balanced scale. Taste was, by far, the most important attribute compared to other attributes in the category ‘very important’. Its mode value was 5 on the five-step scale. Among other attributes, the largest portion of panelists found aroma, color, and thickness of the groundnut–choco spread to be important (Table 1).

**Table 1:** Descriptive statistics of variables in the sample applied in the empirical analysis

Variable	Variable description	Mean	Std. dev.	Mode
***Dependent variables***				
Assur_qual	Intent to pay for quality assurance = 1 if yes	0.15	0.35	0.00
Aflatoxin	Knowledge about aflatoxin = 1 if yes	0.11	0.31	0.00
***Independent variables***				
Likeness^a^	How did you like groundnut–choco spread?	7.10	2.48	9.00
Color^b^		3.50	1.13	4.00
Thickness^b^		3.56	1.02	4.00
Taste^b^		4.10	1.01	5.00
Crunchiness^b^		3.35	1.14	4.00
Size	Preferred groundnut–choco spread package size; 100 g = 1; 250 g = 2; 350 g = 3; 450 g = 4; 550 g = 5; above 550 g = 6	3.13	1.15	3.00
Gender	=1 if male	0.43	0.49	0.00
Age	Age (in years)	29.61	9.57	23.00
Marital status	=1 if panelist is married	0.35	0.47	0.00
Household size	Number of household members including panelist	3.70	2.73	1.00
Less than 18 years	=1 if there are household members younger than 18 years	0.50	0.50	1.00
Income	Household income in month preceding survey in Ghanaian cedis	621.28	619.99	300.00
Education	1 = no formal education; 2 = junior high school/middle school; 3 = senior high/GCE O-A level; 4 = vocational school; 5 = technical school; 6 = teacher training; 7 = nursing training; 8 = university; 9 = postgraduate	5.16	2.66	8.00
Government employee	=1 if panelist is government/civil/public employee	0.44	0.49	0.00

a A nine-step scale ranging from 1 = did not like it at all to 9 = liked it very much and 5 = neither liked nor disliked it.

b Scale: 1 = not important at all; 2 = not important; 3 = neither important nor unimportant; 4 = important; 5 = very important.

Aroma importance (mode 4 on the five-point balanced scale), in particular, stands out and suggests that the combination of groundnut and cocoa is very attractive to panelists. Crunchiness was not expected to be important, and the results show that its mode and median values were 4 on the five-point balanced scale. Crunchiness, although it was ranked relatively high in importance (above 3 on average), was the last in importance among the four attributes and the overall likeability.

From a manufacturer and consumer standpoint, the size of the container of groundnut–choco spread is important because consumers in Ghana have been conditioned by manufacturers of other foods sold in plastic jars or cans to seek a specific container size. The presence of jars of specific size shapes buyer expectations, and manufacturers consider consumer desires seeking a competitive advantage (25). Moreover, if a manufacturer decides to buy packaging equipment, the investment is likely to pay off if the size of containers it handles corresponds to consumer expectations. In Ghana, like in many other sub-Saharan countries (9), small food processors can purchase recycled jars, but such containers pose safety risk. Containers from vendors trading in packaging materials may cost more, and are clean, but they still have to adhere to the size expected by customers. Thus, the question presented to panelists listed six jar sizes. The most preferred among panelists (53%) is the 350-g groundnut–choco spread jar size. The balance was about equally divided between those selecting smaller or larger jar sizes.

Among the untrained panelists, 57% of the panelists were male, 35% were married, and 50% had at least one household member younger than 18 years (Table 2). The average household size comprised 3.7 persons. About 44% of the panelists were government employees, 33% were self-employed, and the remaining 23% were unemployed, retired, or students. The panelists’ age ranged from 18 to 71 years, with an average person being about 30 years old (median age 27 years). The relatively young age is not a surprise, given the demographic structure of the country’s population, where an estimated 57.3% were 24 years old or younger in 2014 (26). Almost 98% of the panelists had formal education, and 41% received a university or postgraduate degree; therefore, the mode value of 8 (university degree) is not surprising. The income reported by panelists ranged from 50 to 5,000 Ghanaian cedi, with the mean of 621 Ghanaian cedi.^1^

**Table 2:** Distribution of responses regarding the importance of attributes of groundnut–choco spread using a five-step scale (in %)

Attribute	Not important at all (%)	Not important (%)	Neutral (%)	Important (%)	Very important (%)
Color	4.06	22.52	9.74	46.65	17.04
Aroma	2.22	18.38	10.10	49.49	19.80
Thickness	2.02	18.18	17.37	46.06	16.36
Taste	1.62	10.73	5.26	40.69	41.70
Crunchiness	5.33	22.75	18.03	38.93	14.96

Figures in rows may not add to 100% due to rounding.

The empirical examination of the intent to pay for quality assurance and knowledge about aflatoxin using regression technique was preceded by the preliminary exploration of the related factors. The analysis was conducted using PROC FACTOR in SAS software, version 14.1. Four factors are associated with paying for quality assurance and knowledge about aflatoxin. Each of the four factor loadings exceeding 0.6 that was associated with either variable consisted of a very similar and distinct set of characteristics. The first factor was associated with the four attributes of the groundnut–choco spread (each loading exceeding 0.75) and the overall likeability of the product; the second factor was positively associated with traditional households characterized by older and married panelists with children aged less than 18 years. In the case of knowing about aflatoxin, this factor also is positively associated with income. Education and knowledge about aflatoxin were identified in factor 3, while with factor 4 two measures were negatively associated: household size and the presence of children aged less than 18 years.

### Statistical validity of logistic relationships

The results in Tables 3 and 4 show the odds ratios, the corresponding *z*-statistics, the marginal effects, and the *P*-values of marginal effects. The odds ratios, where the coefficient is the effect of a unit change in an explanatory variable on the predicted odds ratio while other variables are held constant, carry more practical information than logistic slope coefficients. For example, the odds ratio greater than 1 suggests that the event is more likely to happen than not to happen.

**Table 3:** Logit results identifying factors that affect the intent to pay for quality assurance on groundnut–choco spread label and their individual effects on probability of the intent to pay

Variables	Odds ratio	*z*	Probability change	*P*-value
Liked (categories 7 and 9)	3.938^*^	1.76	0.10^*^	0.08
Color (1 = very important)	2.084	1.36	0.06	0.17
Thickness (1 = very important)	0.518	–0.95	–0.05	0.34
Taste (1 = very important)	0.957	–0.10	–0.00	0.92
Crunchy (1 = very important)	0.407	–1.28	–0.07	0.20
Aroma (1 = very important)	5.987^***^	3.09	0.14^***^	0.00
Gender (male dummy)	1.569	1.24	0.03	0.21
Age (1 = 30 years old or younger)	0.381^**^	–2.12	–0.072^**^	0.03
Low income dummy	1.04	0.10	0.00	0.92
Household size	1.07	0.85	0.01	0.40
Knowledge of aflatoxin (1=yes)	3.614^***^	5.60	0.10^***^	0.00
Marital status (1 = single)	2.450^*^	1.80	0.07^*^	0.07
Children younger than 18 years (1 = yes)	0.438^*^	–1.65	–0.062^*^	0.098
Education	2.501^**^	2.29	0.07^**^	0.02
Jar size (1 = 450 g or larger)	2.709^***^	2.64	0.08^***^	0.01
Constant	0.007^***^	–5.56	-	-
Number of observations	452			
LR Chi^2^ (16)	100.853			
Prob > Chi^2^	0.0000			
Pseudo *R*^2^ (McFadden’s)	0.301			
Tjur’s *R*^2^	0.301			
Log likelihood	–116.93			
Proportion correctly classified	90.49%			

* *P* < 0.1;

** *P* < 0.05;

*** *P* < 0.01.

**Table 4:** Logit results identifying factors characterizing knowledge of aflatoxin and their individual effects on the probability of such knowledge

Variables	Odds ratio	*z*	Probability change	*P*-value
Liked groundnut–choco spread	0.764^***^	–2.98	–0.0240^***^	0.002
Color	0.692	–1.61	–0.0328	0.105
Thickness	1.114	0.33	0.0097	0.743
Taste	2.059^***^	2.75	0.0645^***^	0.005
Crunchiness	1.533^*^	1.66	0.0382^*^	0.094
Education	2.216^*^	1.84	0.0711^*^	0.064
Gender	1.791	1.52	0.0521	0.126
Age	1.015	0.57	0.0013	0.566
Household size	0.914	–0.94	–0.0080	0.347
Marital status	0.699	–0.75	–0.0320	0.453
Children younger than 18 years	2.689^*^	1.93	0.0884^*^	0.052
Government employee	1.890	1.58	0.0569	0.112
Income	1.001^**^	2.44	0.0001^**^	0.013
Constant	0.003	–4.21		
Number of observations	330			
LR Chi^2^	50.57			
Prob > Chi^2^	0.0000			
Pseudo *R*^2^ (McFadden’s)	0.2010			
Tjur’s *R*^2^	0.2077			
Log likelihood	–100.50			
Proportion correctly classified	89.39%			

* *P* < 0.1;

** *P* < 0.05;

*** *P* < 0.01.

The interpretation of odds effects has limited practical value, therefore Tables 3 and 4 report the associated average marginal effects of each predictor on the probability of a positive outcome in dependent variable, that is, intent to pay for quality assurance and knowledge of aflatoxin. The marginal effect of a continuous variable measures change in the probability of dependent variable as the former changes by a unit (e.g. 1 year older than the average age of a panelist). In the case of a binary variable, the average effect shows how the dependent variable changes as the binary variable changes from 0 to 1.

In the case of the intent to pay for quality certification (Table 3), the likelihood ratio Chi^2^, 100.85, is statistically significant, with a *P*-value of 0.0000 suggesting the overall good fit of the specified empirical relationship with the data. The value of pseudo *R*^2^ is 0.301, the same as Tjur’s (Table 3) and common in studies using the cross-sectional data (27). The ability to predict correct outcomes is very high (90.49%; Table 3). Solid predictive performance of both equations is important from practical standpoint of marketing the novel spread by small food processors as they identified statistically significant effects truly influencing consumer choice.

## Discussion

Groundnut paste available in Ghana is often contaminated by aflatoxin as it is in other countries of the region (28) or other parts of sub-Saharan Africa (29, 30), and consumers are not able to assess visually the paste quality (30). Under the circumstances, quality certification is necessary as it results in discarding some of the purchased raw groundnuts during sorting. Product attributes appear to be important in the intent to pay for the quality certification of novel spread, and their quantified effects are quite large (Table 3). Certification required for proper monitoring of the manufacturing process was found important to Ghanaian consumers (31).

Likability is a prominent factor influencing the intention to pay for quality certification. The odds suggest that those who liked the product ranking it high on a nine-point balanced scale were 3.9 times more likely to pay as compared to those who liked the spread less. The probability of choosing to pay increased by 10.3% for panelists who liked the product. It appears that aroma is a critical attribute of the novel groundnut–choco spread since the panelists who felt it was a very important attribute had almost six times higher odds to pay for quality certification. The probability of intending to pay increased by 13.5%, the largest probability change of all statistically significant factors. The combination of roasted groundnut and cocoa appears to have a very strong effect on panelists’ decision.

A panelist with a university or postgraduate degree is 2.5 times more likely to pay extra for a quality assurance label (Table 3) than a panelist with less education. The average marginal effect of education shows that the probability of paying for certification increases by 6.9% for university or postgraduate degree holders (Table 3). Younger panelists had low odds of choosing to pay for quality certification, and being 30 years old or younger lowered the probability by 7.2% as compared to older panelists.

A negative effect on the intent to pay for safety certification is confirmed regarding the presence of children in a household (Table 3). Young children at home are associated with decreasing odds (0.438) of a panelist’s intent to pay for quality assurance (Table 3) as suggested by logistic regression results. The probability of intending to pay for safety certification of the spread associated with the presence of young children at home decreases by 6.2% (Table 3) as compared to panelists not having children. Because the vast majority of households in Ghana have children, the decreased probability of paying for safety certification puts pressure on food manufacturers to price the certified spread, or to market the spread stressing its sensory attributes and likability, among others.

Panelists who know about aflatoxin have 3.6 times higher odds (Table 3) regarding the intent to pay for quality certification as compared to those who do not know about aflatoxin. The high odds are reflected in an increase in probability of the intent to pay for quality certification an extra 9.7% (Table 3).

The size of the jar of spread also affects the intent to pay a premium. A panelist who prefers a jar size of 450 g or larger is 2.7 times more likely to pay for a quality assurance label as compared to smaller size jars (Table 3). Also, the probability of the intent to pay extra for certification increases by 7.5% (Table 3). A larger jar permits the use of a large label to communicate product quality and efforts undertaken to safeguard it; therefore, preference for a larger jar allows educating consumers about the distinctiveness of spread.

The small proportion of panelists who know about aflatoxin (11%; Table 1) is comparable to that of earlier reports from Ghana or other countries in sub-Saharan Africa (e.g. 32–34). Even among groundnut processors and distributors in other countries, only 32% admit knowing about aflatoxin’s detrimental health effects (35). This study quantifies the changes in probability of knowing about aflatoxin with regard to sensory attributes of the novelty spread and socioeconomic and demographic characteristics of panelists and their households. It is presupposed that knowledge about aflatoxin leads to the purchase of quality-certified spread.

If a panelist overall liked the groundnut–choco spread, knowing about aflatoxin is less likely as suggested by the odds value of 0.764 (Table 4). The effect translated into a 2.4% decrease in the probability of knowing about aflatoxin (Table 4). Liking the product could override caution with regard to food safety, and the effect stresses the importance of assuring the supply of safe groundnut products.

The spread’s taste and crunchiness are also important predictors of probability of knowledge about aflatoxin as indicated by logistic regression results (Table 4). Groundnuts are stored in ambient temperatures throughout the marketing chain in Ghana, while many households lack refrigeration to store food products. Ambient temperatures encourage the gradual growth of mold on kernels at high relative humidity levels, and auto-oxidation of groundnut paste is rapid (19), resulting in progression of rancidity and thus altering the taste. A panelist who liked the taste and crunchiness has 2.06 (Table 4) and 1.53 (Table 4) higher odds, respectively, of reporting to know about aflatoxin than those who did not like the taste and crunchiness. Specific attributes are characterized by particularly heavy loading in preliminary factor analysis, and their positive association with knowing about aflatoxin reflects discerning panelists (mold can affect the taste) or is confounded by other factors. The more the panelist likes the overall taste and crunchiness of the spread, the higher the probability of aflatoxin knowledge (6.5% for taste and 3.8% for crunchiness; Table 4).

The study results identify income, education level, and having young children at home as factors increasing the odds, that is, the chances of knowing about aflatoxin increase (Table 4). An increase in income above the average leads to an increase in odds ratio by 1, suggesting that those with high incomes (Table 4) are considerably more likely to be aware of aflatoxin. Accordingly, the marginal effect of income obtained from logistic regression suggests that if the average income increases by 100 cedis (or about 16% above the sample mean value shown in Table 1), the probability of knowing about aflatoxin increases by 1% (Table 4). Per capita income has increased by 15.3% between 2011 and 2015, possibly and indirectly contributing to the effect of income on aflatoxin knowledge.

Higher educational attainment is a likely contributor to wider knowledge and appears specifically to be associated with knowing about aflatoxin as confirmed by the logistic regression results (Table 4); if a panelist has a university or postgraduate degree, he or she is 2.22 times more likely to know about aflatoxin (Table 4). The average marginal effect of education shows that the probability of aflatoxin knowledge increases by 7.1% (Table 4) for university or postgraduate degree holders. Since there has been a rapid increase in college enrolment in recent years, the effect of education is likely to improve consumer knowledge about aflatoxin.

The presence of children and greater odds of knowledge of aflatoxin are highly encouraging results confirmed by logistic regression (Table 4) because children are particularly vulnerable to the effects of aflatoxin (5). Specifically, panelists who have young children at home are 2.69 times more likely to know about aflatoxin than those with no young children (Table 4). The increase in probability of knowing about aflatoxin increases by 8.8% if young children are present at home (Table 4).

## Conclusion

Groundnuts and groundnut products are consumed daily by many residents of Ghana and sub-Saharan Africa. In Ghana, the existing standards do not differ from those in many other countries but are excluded from compliance by numerous micro and small enterprises. Consequently, processors are free to decide whether to use only pre-sorted groundnuts that, in turn, may impact the extent to which groundnut products are safe from aflatoxin.

Food processors are forced to supply safe groundnut products (21, 36). This study, using data obtained from untrained panelists expressing their subjective opinions, shows that panelists who express intent to pay for quality certification readily accept a novel groundnut–choco spread. A domestic food manufacturer using local ingredients can offer a novel groundnut spread that promises success in marketplace. The manufacturing process assuring safety is a matter of concern for Ghanaian consumers (31).

Results of the current study assign changes in probabilities to likability, sensory attributes, and panelist characteristics associated with preference of quality certification. For groundnut processors, the key message to communicate to buyers is that the production process adheres to procedures that assure that only safe ingredients are used in the final product. Interestingly, the current study’s findings that both household size and number of children aged less than 18 years lower the probability of choosing to pay for quality certification coincide with similar variables associated with high levels of aflatoxin in blood samples among Ghanaians (37). The effect of sensory attributes, especially aroma, seems critical for the groundnut–choco spread in encouraging purchase. This may require in-store sampling opportunities in promoting sales of the quality-certified product.

Provision of appropriate packaging material helps small enterprises to enter formal markets (9). Panelists expressing preference for a 450-g or larger jar have more than 7.5% higher probability of choosing to pay for quality assurance on product label. The jar size allows placing a sufficiently larger label with information of why and how safety certification is accomplished. Lowering the costs of quality certification has been suggested among the development of a system of licensed laboratories. Many small enterprises fail because of nonfinancial reasons (13), but food scientists could offer guidance about specific information that can be placed on labels. However, such assistance is not yet readily available in Ghana because the links between the industry and researchers are incidental due to enterprises’ limited resources.

Such situation opens opportunities for the government agencies to develop certification procedures, and implementing them in the initial stages facilitates access to laboratories of government research institutes. Initially, the government may choose to subsidize the certification of spread for micro and small enterprises to support the market entry of their product. Gradually, the government could shift certification to commercial laboratories while monitoring their performance, pricing of service, and assuring consistently high-quality certification services. The approach would contribute to the broadening of the role of private sector in emerging food sector and may establish grounds for wide retailing and even exporting of certified spread. Retailers in sub-Saharan Africa consider certification of products while seeking suppliers (38).

Separately, the government could prepare a framework allowing university scientists to formally assist private micro and small enterprises in the scale-up of novelty-spread manufacturing. Such services, available in the developed countries, have proved important in accelerating the proportion and, consequently, reducing the costs and increasing the flow of revenues of enterprises. Participation of nongovernmental organizations (NGOs) in some of the activities, including training, could be helpful.

## Footnotes

1 Around USD 320 in August 2012.
